# The Anti-Rheumatic Drug, Leflunomide, Induces Nephrotoxicity in Mice via Upregulation of TGFβ-Mediated p53/Smad2/3 Signaling

**DOI:** 10.3390/toxics10050274

**Published:** 2022-05-23

**Authors:** Alhanouf A. Aljohani, Yasmeen S. Alqarni, Maram N. Alrashidi, Maha H. Aljuhani, Shaimaa A. Shehata, Mohamed K. El-Kherbetawy, Kousalya Prabahar, Reem Alshaman, Abdullah Alattar, Ahmed M. N. Helaly, Hayam Ateyya, Ezzat A. Ismail, Sawsan A. Zaitone

**Affiliations:** 1 Pharm D Program, Faculty of Pharmacy, University of Tabuk, Tabuk 71491, Saudi Arabia; 381011107@stu.ut.edu.sa (A.A.A.); 381008151@stu.ut.edu.sa (Y.S.A.); 371000282@stu.ut.edu.sa (M.N.A.); 371003833@stu.ut.edu.sa (M.H.A.); 2 Department of Forensic Medicine and Clinical Toxicology, Faculty of Medicine, Suez Canal University, Ismailia 41522, Egypt; shaimaa_shehata@med.suez.edu.eg; 3 Department of Pathology, Faculty of Medicine, Suez Canal University, Ismailia 41522, Egypt; mohamed_elkherbetawy@med.suez.edu.eg; 4 Department of Pharmacy Practice, Faculty of Pharmacy, University of Tabuk, Tabuk 71491, Saudi Arabia; kgopal@ut.edu.sa; 5 Department of Pharmacology & Toxicology, Faculty of Pharmacy, University of Tabuk, Tabuk 71491, Saudi Arabia; ralshaman@ut.edu.sa (R.A.); aalattar@ut.edu.sa (A.A.); 6 Department of Forensic Medicine and Toxicology, Faculty of Medicine, Mansoura University, Mansoura 35516, Egypt; ahmedhelaly@mans.edu.eg; 7 Clinical Science, Faculty of Medicine, Yarmouk University, Irbid 566, Jordan; 8 Department of Medical Pharmacology, Faculty of Medicine, Cairo University, Giza 11559, Egypt; hayamatya@cu.edu.eg or; 9 Department of Pharmacy Practice and Clinical Pharmacy, Faculty of Pharmacy, Future University in Egypt, Cairo 11835, Egypt; 10 Department of Urology, Faculty of Medicine, Suez Canal University, Ismailia 41522, Egypt; boezzat@med.suez.edu.eg; 11 Department of Pharmacology & Toxicology, Faculty of Pharmacy, Suez Canal University, Ismailia 41522, Egypt

**Keywords:** leflunomide, mice, nephrotoxicity, p53/Smad2/3 pathway, TGFβ fibrosis

## Abstract

Recent studies indicated renal toxicity and interstitial nephritis in patients receiving leflunomide (LEFN), but the exact mechanism is still unknown. The transforming growth factor β (TGFβ)/p53/Smad2/3 pathway crucially mediates renal fibrosis. We aimed to assess the nephrotoxic effect of LEFN in mice and the possible role of TGFβ-stimulated p53/SMAD2/3 signaling. The study design involved distributing sixty male albino mice into four groups: (i) vehicle-treated mice, (ii) LEFN (2.5 mg/kg), (iii) LEFN (5 mg/kg), and (iv) LEFN (10 mg/kg). The drug was given orally every 48 h and continued for 8 weeks. Blood samples were then taken from mice for the determination of kidney function parameters. Right kidneys were used for histopathologic staining and immunohistochemistry, whereas left kidneys were frozen and used for Western blot analysis of the target proteins, p-p53 and Smad2/3. Results indicated that chronic administration of LEFN in mice resulted in a four- and nine-fold increase in serum urea and creatinine levels, respectively. Kidney specimens stained with hematoxylin and eosin or periodic acid–Schiff showed significant histopathological manifestations, such as cellular irregularity, interstitial congestion, and moderate lymphocytic inflammatory infiltrate in mice treated with LEFN. Western blotting indicated upregulation of the p-p53/Smad2/3 proteins. LEFN, especially in the highest dose (10 mg/kg), produced prominent nephrotoxicity in mice. This toxicity is mediated through stimulating fibrotic changes through TGFβ-stimulated p53/Smad2/3 signaling and induction of glomerular and tubular apoptosis. An improved understanding of LEFN-induced nephrotoxicity would have great implications in the prediction, prevention, and management of leflunomide-treated rheumatic patients, and may warrant further clinical studies for following up these toxidromes.

## 1. Introduction

Leflunomide (LEFN) is an anti-cellular proliferation and immunomodulator drug that is used in rheumatoid arthritis treatment, as a disease-modifying anti-rheumatic drug (DMARD), and in numerous autoimmune diseases [1]. LEFN is completely metabolized into teriflunomide (A-771726): an active metabolite in the liver and gut mucosa [2]. LEFN has been proven to induce hepatotoxicity, anaphylaxis, toxic epidermal necrolysis, Stevens–Johnson syndrome, and pulmonary effects [3,4,5,6,7]. Additionally, hepatotoxicity induced by LEFN was linked to mitochondrial dysfunction at the transcript level [8], oxidative stress [9,10], and unfolded proteins accumulation [11]. In a recent study, LEFN induced inflammatory reactions and pulmonary toxicity in mice, together with architectural structural distortion [12].

LEFN is not known as a nephrotoxic agent; however, emerging evidence from numerous case reports proved that LEFN induces nephrotoxicity (chronic interstitial nephritis), even with the recommended dose of leflunomide [13,14,15,16,17]. The kidney is a vulnerable vital organ in drug elimination; therefore, chemical exposure to drugs and their metabolites will jeopardize the urinary tract system [18,19]. Toxic damage (either acute or chronic) to the kidney is attributed to various substances, such as hydrocarbons, heavy metals, and proven nephrotoxic drugs, as well as aflatoxins that might result in renal cancer or failure [20,21,22,23].

The pathogenic processes of LEFN-induced nephrotoxicity are still unknown. In fact, drug-induced renal injury can be attributed to numerous pathogenic pathways [24]. Transforming growth factor-β (TGFβ) is a pleiotropic cytokine accountable for the regulation of several important cellular pathways and activities throughout the human body. Ordinarily, TGFβ exerts a pivotal function in maintaining tissue homeostasis largely via gene transcriptional regulation, which is responsible for cell proliferation, cytostasis, and survival [25,26,27,28]. The binding of a TGFβ isoform to a heterotetrameric complex of the serine/threonine kinase receptors is the key factor in TGFβ signaling propagation [29]. Activating the TGFβ receptors induces transcription factors Smad2/3 phosphorylation downstream [30,31,32]. Consequently, the activated Smad complex storage in the nucleus increases, which plays an essential role in the regulation of transcription of several target genes [33,34].

TGFβ is a key factor in triggering renal fibrosis in a variety of chronic kidney diseases. Moreover, TGFβ overexpression has been observed in the advanced stages of renal disease [35,36]. The activation of the TGFβ–Smad complex signal results in stimulation of myofibroblasts, extreme generation of extracellular matrix (ECM), and inhibition of ECM degradation [35].

The tumor suppressor gene (p53), known as the “Guardian of the Genome”, plays a significant role in the regulation of nuclear protein gene transcription and encoding. These genes are responsible for cellular apoptosis and cell-cycle arrest [37,38,39]. The p53 response may be prompted by numerous stress triggers, such as oncogene activation, genotoxic agents, hypoxia, and ribosomal stress [40]. The last four decades have seen a growing knowledge of p53 function as an oncogene targeting cancer cells. Furthermore, mutation of TP53, the gene encoding for p53, is the most frequently mutated gene among cancer patients [41,42,43]. Indeed, p53 gene overexpression is noted in several cancer types [39].

Under normal conditions, activated p53 and TGFβ act as gene-specific transcription factors, assisting in the control of multiple genes required in the generation of antitumor effects [44]. Due to the wide alternating nature of these proteins, overlap of cellular functions occurs in the regulation of autophagy and apoptosis, indicating several possible ideas of convergence. Wyllie et al. (1991) suggested a link between the TGFβ and wild-type p53 pathway, stating that wild-type p53 deactivation by the SV40 virus was reported to result in losing the reaction to TGFβ treatment, indicating that p53 loss may initiate resistance to the anticancer effects of TGFβ [45]. Various studies have established the role of TGFβ1/p53 signaling in the generation of renal fibrosis [46]. Furthermore, TGFβ promotes the activation of the p53 and Smad complexes, both of which play important roles in renal fibrogenesis [46].

Hence, our aim was to assess LEFN-induced dose-dependent renal toxicity or fibrogenesis in mice, and the possible role of TGFβ-stimulated p53 apoptosis.

## 2. Materials and Methods

### 2.1. Signaling Pathway Enrichment Analysis

The chosen target pathway was selected utilizing online databases such as KEGG pathway [47] and Reactome [48].

### 2.2. Animals

This experiment was performed using sixty male Swiss albino mice. Mice were housed in a normal light/dark cycle and a temperature equal to 25 ± 5 °C, with free access to chow diet and water. Mice were acclimatized to the experimental conditions in the animal house for seven days prior to the experiment. This study protocol was approved by the Institutional Ethics Committee (201603A7c).

### 2.3. Design of the Experiment

Four groups of mice were used, with fifteen mice each. LEFN was prepared as a suspension with the aid of 1% carboxymethylcellulose (CMC) solution. Oral gavaging of LEFN continued for 8 weeks according to the study design: vehicle group: mice received 1% CMC solution; LEFN 2.5 mg/kg group: mice received a dose equal to 2.5 mg per kg; LEFN 5 mg/kg group: mice received a dose equal to 5 mg per kg; and LEFN 10 mg/kg group: mice received a dose equal to 10 mg per kg. For all groups, drug or vehicle administration by oral gavage was performed every 48 h and continued for 8 weeks.

### 2.4. Collection of Blood Samples and Kidneys

After finishing the course of LEFN, blood was collected under ketamine anesthesia (80 mg per kg of ketamine HCL) by intraperitoneal injection [49,50]. Blood was collected in tubes, and after standing for 25 min at room temperature, samples were centrifuged at 1600× *g* for 10 min. Serum samples were then kept at −20 °C until used for renal estimation of function parameters. The right kidneys were frozen immediately at −80 °C.

### 2.5. Estimation of Renal Function Parameters

The quantification of serum creatinine was performed applying the alkaline-picric acid method according to the method described in the kit instructions. The quantification of urea was achieved via applying the reaction with salicylate-hypochlorite in the presence of nitroprusside; this reaction yield forms a green indophenol product. Kits were purchased from Diamond Diagnostics Ltd. (Cairo, Egypt). A UV1601-PC spectrophotometer (Schimadzu, Kyoto, Japan) was used to quantify the optical density of the reaction products.

### 2.6. Western Blot Analysis for p-p53 and SMAD2/3 in the Renal Homogenate

As a basic step, we determined the total protein concentration in 5-µL samples of the kidney homogenates. We used a Bio-Rad Quick StartTM Bradford Protein Assay kit (Hercules, western Contra Costa, CA, USA). Equal protein amounts from the kidney homogenates were denaturated using 4× Laemmli sample buffer from Bio-Rad, and then subjected to SDS polyacrylamide gel electrophoresis for separation of the proteins. Then, we transferred the proteins to a nitrocellulose membrane and blocked the free sites on the membranes by incubation for 1 h in Bio-Rad non-fat dried milk. After that, we washed the membranes and incubated them with primary antibodies for p-p53 (ab33889), smad2/3 (202445), and β-actin (Ab8226) (Abcam, Waltham, MA, USA) overnight. Then, we washed the blots and added secondary antibodies conjugated with horseradish peroxidase. An Amersham BioSciences chemiluminescence kit (Buckinghamshire, UK) was used to detect the concentration of each protein. Then, the blots were imaged, and the band densities were measured by ImageJ software (NIH, Bethesda, MD, USA).

### 2.7. Kidney Histopathology and Immunohistochemistry

The left kidneys were fixed in 10% phosphate-buffered formalin. After standing overnight, tissues were dehydrated and embedded in paraffin. Then, serial sections were cut to 4-μm and stained with hematoxylin and eosin (H&E), and periodic acid–Schiff (PAS) stain for the detection of mucopolysaccharides at the glomerular capillary basement membranes [51]. Slides were also stained with Masson’s trichrome for the detection of collagen deposition. A blinded experienced pathologist examined the stained sections under a light microscope (Olympus^®^ CX21) and captured several representing images. The pathologist captured images using a calibrated digital microscope camera (Tucsen ISH1000 digital microscope camera) with 10 megapixels resolution (3656 × 2740 pixel each image) at original magnifications equal to 100× and 400× (objective lens 10× and 40×), and a UIS optical system (Universal Infinity System, Olympus^®^, Tokyo, Japan). We also used the “IS Capture” software to enhance the images. Kidney slides were captured.

#### 2.7.1. Morphometric Analysis of H&E-Stained Sections

Slides were examined for the pathological changes described (lymphocytic infiltrate, congestion, and hemorrhage, and tubular and glomerular degeneration), and the severity of changes was assigned using scores on a scale of 0–5 (score (0) denotes no change, score (1) is changes affecting < 20%, score (2) is changes affecting 20 −< 40%, score (3) is changes affecting 40 −< 60%, score (4) is changes affecting 60 −< 80%, score (5) is changes affecting 80–100%) and applied for five readings per group [52].

#### 2.7.2. Examination of PAS-Stained Sections

For the quantification of integrated intensities (pixels) and area percentages (%) of mucopolysaccharides using periodic acid–Schiff (PAS) stain, twenty-five captured microscopic images at 400× magnification fields were evaluated using ImageJ software. All positive orange-to-red reactions were captured in different groups. Random fields were selected in the control non-treated group using ImageJ software [53]. Slides were examined for pathological changes, described as: lymphocytic infiltrate, congestion and hemorrhage, and tubular and glomerular degeneration. These changes were assigned on a scale of none = 0, mild (changes affecting < 25%) = 1, moderate (changes affecting 25–50%) = 2, and severe damage (changes affecting > 50%) = 3 [54].

### 2.8. Immunohistochemistry and Image Analysis of TGFβ and p53 in Renal Tissues

Serial sections were deparaffinized, hydrated, and then immersed in EDTA solution (pH = 8) for antigen retrieval. Hydrogen peroxide (0.3%) was then added to the slides for 12 min, and then antibodies to p53 (GTX100629, 1:500, Irvine, GeneTex, CA, USA) and TGFβ1 (GTX45121, 1:100, Irvine, GeneTex, CA, USA) were applied at 4 °C for 12 h. Phosphate buffered saline (PBS) was used to wash the slides three times, and then the secondary antibodies were applied to the slides for 1 h. A Power-StainTM 1.0 poly HRP-3,3-diaminobenzidine tetrachloride (DAB) kit (Genemed Biotechnologies, South San Francisco, CA, USA) was used to detect the color of the reaction, and Mayer’s hematoxylin was applied as a final stain. Multiple fields were imaged and analyzed using the ImageJ program (NIH, Bethesda, MD, USA).

### 2.9. Statistical Analysis

We tabulated and averaged the whole data. In case of quantitative data with normal distribution, we applied one-way ANOVA, whereas post hoc analysis was performed by Bonferroni’s test. The scoring data were presented as medians and quartiles, and analyzed by the Kruskal–Wallis ANOVA and Dunn’s test. The bilateral significance was set at α = 0.05.

## 3. Results

### 3.1. The Bioinformatic Study

**Figure 1:**
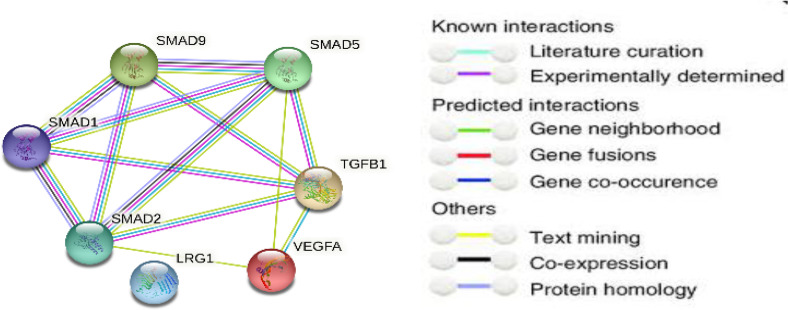
Network protein–protein interaction analysis for TGFβ and Smad proteins as presented by the STRING database.

**Figure 2:**
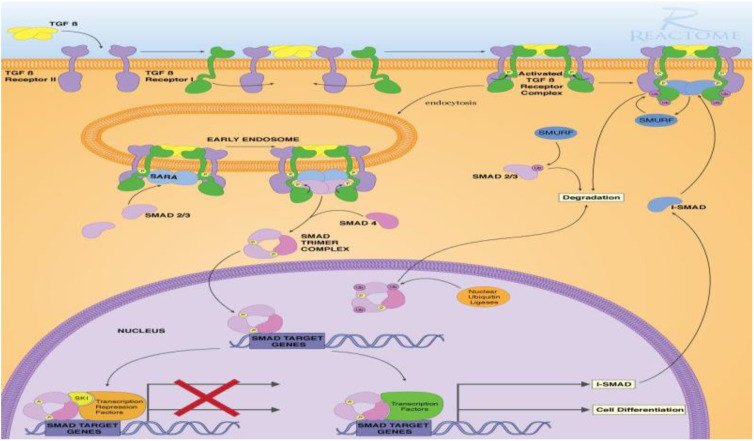
TGFβ-induced Smad signaling, created via Reactome and KEGG bioinformatic data bases.

For highlighting the role of the TGFβ signaling pathways and demonstrating the key players in its signal transduction, bioinformatic analysis was performed utilizing the STRING database, which is a comprehensive database. It shows the predicted interaction between proteins, and reveals whether this is a physical (direct) or indirect interaction. In the STRING database, protein–protein interactions are given a “score” individually; this is done according to seven evidence channels which are marked by colored lines or edges in Figure 1, representing experimental evidence, database citations, gene neighborhooding, fusions, as well as co-occurrence, appearance in literature text, co-expression, and protein h. The bioinformatic tools demonstrated the ability of TGFβ to activate Smad proteins. TGFβ activates a transcriptional cascade, which leads, finally, to enhancing the production of Smad proteins (Figure 2).

### 3.2. Assessment of Renal Function

**Figure 3:**
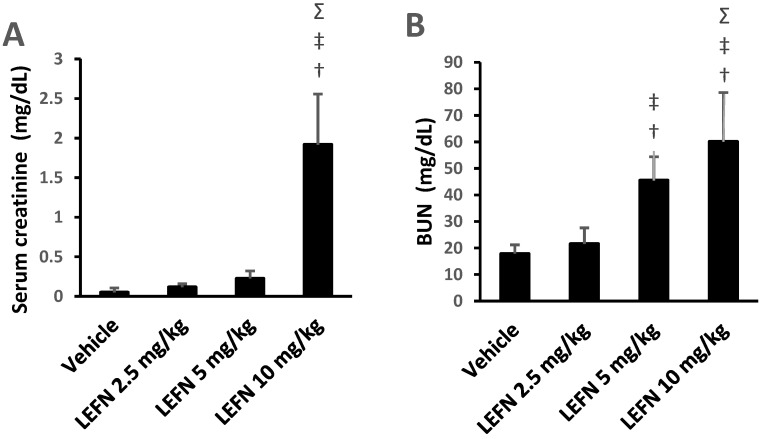
Effect of LEFN on kidney function parameters in adult mice. (**A**) Serum creatinine, (**B**) BUN. Data are mean ± SD, and analysis was performed by applying one-way ANOVA and Bonferroni’s test at *p* < 0.05. †: versus the vehicle group, ‡: versus the LEFN 2.5 mg/kg group, and Σ: versus the LEFN 5 mg/kg group. BUN: blood urea nitrogen.

Figure 3A,B shows the effects of eight weeks oral administration of LEFN (2.5, 5, and 10 mg/kg) on serum creatinine and BUN levels of adult mice. The LEFN (10 mg/kg) group (iv) demonstrated a statistically significant rise in serum creatinine and BUN levels compared with the vehicle group and mice who received LEFN (2.5 or 5 mg/kg) (Figure 3A,B). The LEFN (5 mg/kg) group (iii) displayed a statistically significant high level of BUN compared to groups i and ii Figure 3B.

### 3.3. Kidney Histopathology for H&E-Stained Sections and Scoring

**Figure 4:**
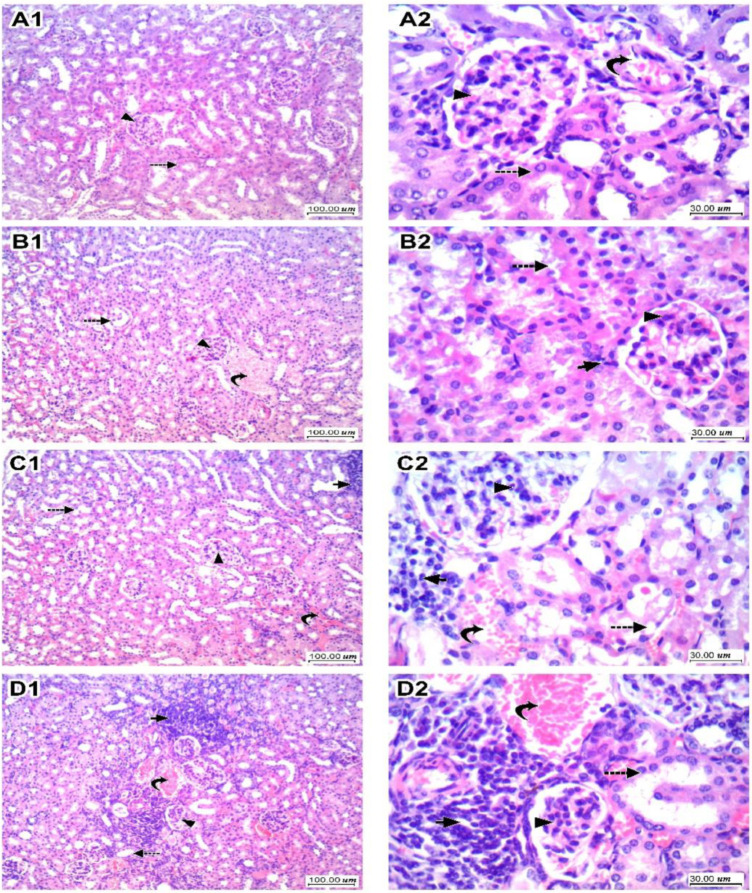
Histopathologic scores for hematoxylin and eosin-stained kidney specimens in mice treated with LEFN. Panel (**A1**,**A2**) show sections of kidney tissue in the vehicle group, showing regular tubules (dashed arrow) lined by epithelial cells with intact eosinophilic cytoplasm and regular nuclei, and glomeruli (arrowhead) showing capillary tuft with a thin wall and thin patent Bowman’s space and mesangial cells. Interstitium showed thin blood vessels (curved arrow) and loose intervening stroma. Panel (**B1**,**B2**) are kidneys from the LEFN 2.5 mg/kg group showing mild focal tubular (dashed arrow) hydropic degeneration of tubular epithelial cells, glomeruli (arrowhead) showing average normal cellularity, and stroma showing focal minimal lymphocytic inflammatory infiltrate (arrow). Panel (**C1**,**C2**) are for kidneys in the LEFN 5 mg/kg group showing moderate tubular (dashed arrow) hydropic and vacuolar degeneration of tubular epithelial cells, glomeruli (arrowhead) showing a slight increase in cellularity, and stroma showing focal mild congestion (curved arrow) and focal mild lymphocytic inflammatory infiltrate (arrow). Panel (**D1**,**D2**), the LEFN 10 mg/kg group showed moderate hydropic degeneration of tubular epithelial cells (dashed arrow), and glomeruli showed a moderate increase in cellularity with irregularity and focal shrinkage (arrowhead). There is interstitial congestion and focal hemorrhage (curved arrow) with moderate lymphocytic inflammatory infiltrate (arrow), ×100 and ×400, respectively.

In Figure 4, sections of kidney tissues in the vehicle group showed regular tubules lined by epithelial cells with intact eosinophilic cytoplasm and regular nuclei, and glomeruli showed capillary tuft with a thin wall and thin patent Bowman’s space and mesangial cells. The interstitium showed thin blood vessels and loose intervening stroma (Panel A1,A2). The LEFN 2.5 mg/kg group showed mild focal tubular hydropic degeneration of tubular epithelial cells, glomeruli showed average normal cellularity, and stroma showed focal minimal lymphocytic inflammatory infiltrate (Panel B1,B2). The LEFN 5 mg/kg group showed moderate tubular hydropic and vacuolar degeneration of tubular epithelial cells, glomeruli showed a slight increase in cellularity, and stroma showed focal mild congestion and focal mild lymphocytic inflammatory infiltrate (Panel C1,C2). Finally, the LEFN 10 mg/kg group showed moderate hydropic degeneration of tubular epithelial cells, and glomeruli showed a moderate increase in cellularity with irregularity and focal shrinkage. There is interstitial congestion and focal hemorrhage with moderate lymphocytic inflammatory infiltrate (Panel D1,D2).

**Figure 5:**
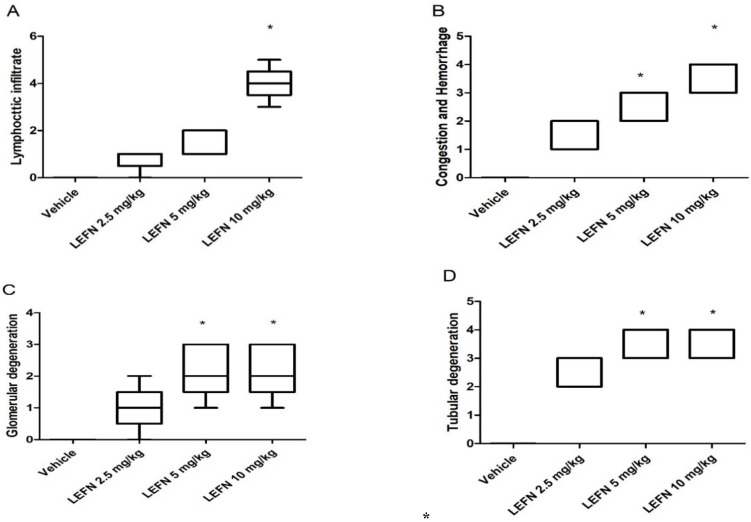
Histopathologic scores for hematoxylin and eosin-stained kidney specimens in mice treated with LEFN. Scores for (**A**) lymphocytic infiltrate, (**B**) congestion and hemorrhage, (**C**) glomerular degeneration, and (**D**) tubular degeneration. A score from 0–5 was given to each sample, and scores are presented as medians and quartiles, and compared by Kruskal–Wallis ANOVA and Dunn’s test at *p* < 0.05. *: versus the vehicle group.

Figure 5 shows the four panels that indicate the histopathologic scores for the H&E-stained kidney specimens. These panels show lymphocytic infiltration (Figure 5A), congestion and hemorrhage (Figure 5B), glomerular degeneration (Figure 5C), and tubular degeneration (Figure 5D). It is shown that the LEFN 10 mg/kg group showed the highest scores, which were significantly greater than the score of the vehicle group every time.

### 3.4. Kidney Histopathology in PAS Stained Sections

**Figure 6:**
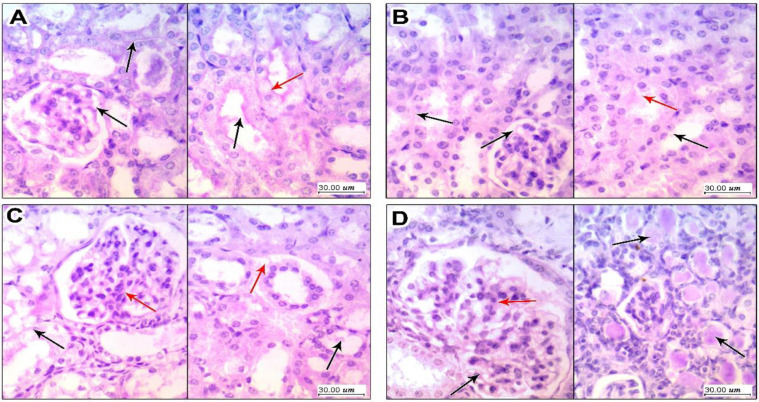
Periodic acid–Schiff staining for the kidney specimen. (**A**) Sections in kidney tissue from vehicle group showing (left panel) glomerulus with thin basement membranes (black arrow) and patent vascular lumen, and tubules showing (right panel) a preserved brush border (black arrow) of tubular epithelial cells with average cytoplasm (red arrow). (**B**) Sections in kidney tissue from the LEFN 2.5 mg/kg group showing (left panel) glomerulus with thin basement membranes (black arrow) and patent vascular lumen, and tubules showing (right panel) a focally disrupted and effaced brush border (black arrow) of tubular epithelial cells and early cytoplasmic vacuoles (red arrow). (**C**) Sections in kidney tissue from the LEFN 5 mg/kg group showing (left panel) glomerular irregularities of vascular lumens with focal increased cellularity (red arrow), but with thin basement membranes (black arrow) (right panel) showing a disrupted and effaced brush border (black arrow) of tubular epithelial cells with moderate cytoplasmic vacuolation (red arrow). (**D**) Sections in kidney tissue from the LEFN 10 mg/kg group showing (left panel) a mild glomerular increase in cellularity (red arrow) with thin basement membranes (black arrow); the right panel shows multiple intratubular eosinophilic hyaline casts filling tubules (black arrow), with marked disruption of lining epithelial cells’ brush borders.

Results of periodic acid–Schiff staining are shown in Figure 6. Kidney specimens in the vehicle group sowed glomerulus with thin basement membranes and patent vascular lumen, and tubules showed (right panel) a preserved brush border of tubular epithelial cells (Figure 6A). Sections in kidney tissue from the LEFN 2.5 mg/kg group showed glomerulus with thin basement membranes and patent vascular lumen, and tubules showed focal disruption and an effaced brush border of tubular epithelial cells (Figure 6B). Sections in kidney tissue from the LEFN 5 mg/kg group showed glomerular irregularities of vascular lumens with focal increased cellularity, but thin basement membranes showed a disrupted and effaced brush border of tubular epithelial cells with moderate cytoplasmic vacuolation (Figure 6C). Sections in kidney tissue from the LEFN 10 mg/kg group showed a mild glomerular increase in cellularity with thin basement membranes, and showed multiple intratubular eosinophilic hyaline casts filling tubules, with a marked disruption of lining epithelial cells’ brush borders (Figure 6D).

### 3.5. Kidney Histopathology in Masson’s Trichrome Stained Sections

**Figure 7:**
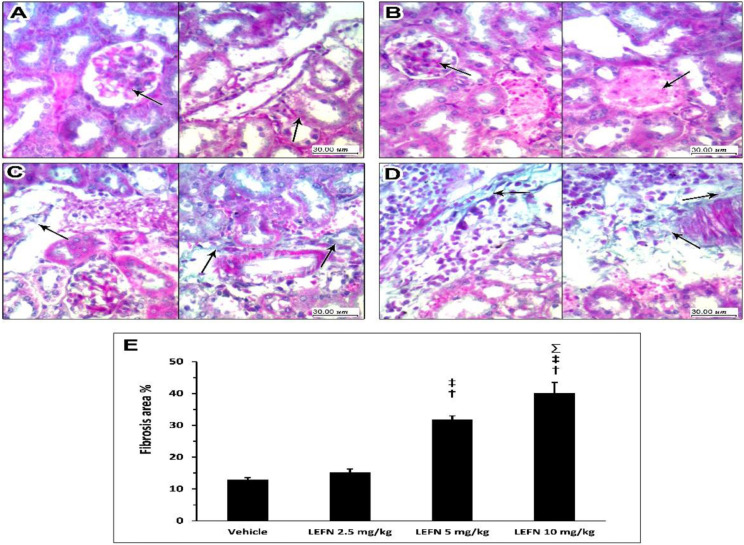
Masson’s trichrome staining for the kidneys of mice treated with LEFN. (**A**, left panel) Sections in kidney tissue from the vehicle group showing glomerulus and proximal tubules with interstitial tissue showing no deposition of green staining fibers in between, and no fibrosis. (**A**, right panel) shows distal tubules and a vessel in the center with no perivascular or peritubular collagen deposition (arrow). Sections from the kidneys of the LEFN 2.5 mg/kg group showing (**B**, left panel) no glomerular deposition of collagen fibers (arrow) (**B**, right panel) shows areas of congested vessels (arrow) with no or faint perivascular green staining, indicating no fibrosis from the kidneys of the LEFN 2.5 mg/kg group. (**C**, left panel) Sections in kidney tissues from the LEFN 5 mg/kg group showing peritubular and interstitial mild focal deposition of thin green staining collagen fibers (arrow). (**C**, right panel) shows areas of perivascular, green-stained collagen fibers deposition (arrow), indicating more localization to perivascular areas in the kidneys of the LEFN 5 mg/kg group. (**D**, left panel) Sections in kidney tissue from the LEFN 10 mg/kg group showing peritubular and interstitial inflammatory infiltrate with deposition of thin green staining collagen fibers (arrow). (**D**, right panel) shows wider areas of perivascular, green-stained collagen fibers deposition (arrow), indicating more localization to perivascular areas from the kidneys of the LEFN 10 mg/kg group. (**E**) Column chart for the mean ± SD of the fibrosis area % as measured in each group kidney sepecimens. †: versus the vehicle group, ‡: versus the LEFN 2.5 mg/kg group, and Σ: versus LEFN the 5 mg/kg group at *p* < 0.05.

Figure 7 shows the Masson’s trichrome staining for the kidney specimens. Sections in kidney tissue from the vehicle group showed (Figure 7A, left panel) glomerulus and proximal tubules with interstitial tissue showing no deposition of green staining fibers in between and no fibrosis; Figure 7A (right panel) shows distal tubules and a vessel in the center with no perivascular or peritubular deposition. Sections from the kidneys of the LEFN 2.5 mg/kg group showed (Figure 7B, left panel) no deposition of collagen fibers, as indicated by faint green staining in peritubular and interstitial areas (arrow). The right panel shows areas of congested vessels with no or faint perivascular green staining, indicating no fibrosis (Figure 7B). Sections in kidney tissues from the LEFN 5 mg/kg group showed (Figure 7C, left panel) peritubular and interstitial mild focal deposition of thin green staining collagen fibers. The right panel shows areas of perivascular green-stained collagen fibers deposition, indicating more localization to perivascular areas (Figure 7C, right panel). Sections in kidney tissue from the LEFN 10 mg/kg group (left panel) showed peritubular and interstitial inflammatory infiltrate with deposition of thin green staining collagen fibers (Figure 7D, left panel). Right panel shows wider areas of perivascular green-stained collagen fibers deposition, indicating more localization to perivascular areas (Figure 7D, right panel).

### 3.6. Immunohistochemical and Image Analysis of TGFβ and p53 Expression in Renal Specimen

**Figure 8:**
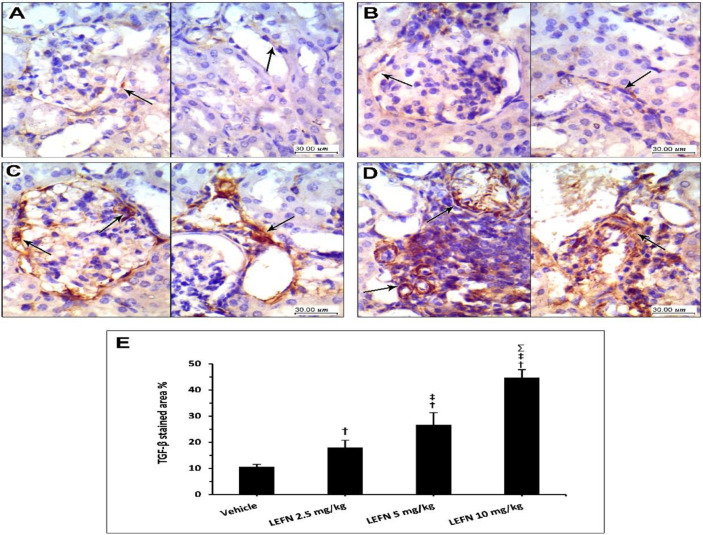
Immunohistochemistry for TGFβ in kidney specimens from LEFN-treated mice. (**A**) Sections from the vehicle group show focal weak TGFβ-staining in normal kidneys, localized to periglomerular and peritubular areas (in left panel), and focal minimal staining in perivascular areas (right panel). (**B**) Sections from the LEFN 2.5 mg/kg group show faint focal staining of TGFβ in periglomerular areas (left panel) and minimal focal weak staining in peritubular areas (arrow in right panel). (**C**) Sections from the LEFN 5 mg/kg group show moderate staining of TGFβ encircling periglomerular areas (left panel), and this is also seen in perivascular areas or focally in peritubular areas (right panel). (**D**) Sections from the LEFN 10 mg/kg group show moderate to strong staining of TGFβ, especially in areas surrounded by inflammatory cells infiltrate (left panel), and perivascular and peritubular areas (right panel), arrows in all images indicate positive staining. (**E**) Column chart for mean ± SD of the stained area % in kidney specimens from the experimental groups. †: versus the vehicle group, ‡: versus the LEFN 2.5 mg/kg group, and Σ: versus the LEFN 5 mg/kg group at *p* < 0.05.

Figure 8 shows the immunohistochemistry for TGFβ in the kidney specimens. Sections from the vehicle group show focal weak TGFβ staining in the normal kidneys, localized to periglomerular and peritubular areas (A, left panel) and focal minimal staining in perivascular areas (A, right panel). Sections from the LEFN 2.5 mg/kg group show faint focal staining of TGFβ in periglomerular areas (B, left panel) and minimal focal weak staining in peritubular areas (B, arrow in right panel). Sections from the LEFN 5 mg/kg group show moderate staining of TGFβ encircling periglomerular areas (C, left panel), and this is also seen in perivascular areas or focally in peritubular areas (C, right panel). Sections from the LEFN 10 mg/kg group show moderate to strong staining of TGFβ, especially in areas surrounded by inflammatory cells infiltrate (D, left panel), and perivascular and peritubular areas (D, right panel).

**Figure 9:**
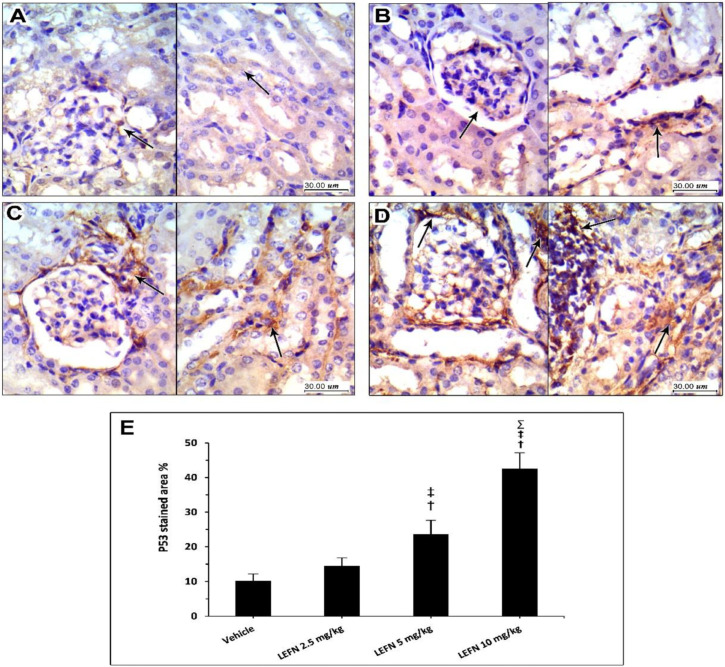
Immunohistochemistry for p53 in the kidney specimens. (**A**) Images from the vehicle group show focal weak p53 staining, localized to periglomerular cells (in left panel) and focal minimal staining in tubular cells (right panel). (**B**) Images from the LEFN 2.5 mg/kg group show a very faint focal staining of p53 in glomerular areas (left panel) and weak staining in peritubular areas (arrow in right panel). (**C**) The LEFN 5 mg/kg group showed focal moderate or weaker staining of p53 encircling periglomerular areas (left panel), and this is also seen in peritubular and perivascular areas (right panel). (**D**) The LEFN 10 mg/kg group revealed moderate to strong staining of p53, with most staining in periglomerular tubules (left panel) and in areas surrounded by inflammatory cells infiltrate (right panel), arrows in all images indicate positive staining (**E**) Column chart for mean ± SD of the stained area % in kidney specimens from the experimental groups and data were analyzed using one-way ANOVA. †: versus the vehicle group, ‡: versus the LEFN 2.5 mg/kg group, and Σ: versus the LEFN 5 mg/kg group at *p* < 0.05.

Figure 9 shows the immunohistochemistry for p53 in the kidney specimens. Sections from the vehicle group show focal weak p53 staining, localized to periglomerular cells (A, left panel), and focal minimal staining in tubular cells (A, right panel). The LEFN 2.5 mg/kg group showed a very faint focal staining of p53 in glomerular areas (B, left panel), and weak staining in peritubular areas (B, arrow in right panel). The LEFN 5 mg/kg group showed focal moderate or weaker staining of p53 encircling periglomerular areas (C, left panel), and this was also seen in peritubular and perivascular areas (C, right panel). The LEFN 10 mg/kg group revealed moderate to strong staining of p53, with most staining in periglomerular tubules (Figure 9D, left panel) and in areas surrounded by inflammatory cells infiltrate (Figure 9D, right panel).

### 3.7. Western Blot Analysis of p-p53 and SMAD2/3 in Renal Homogenates

**Figure 10:**
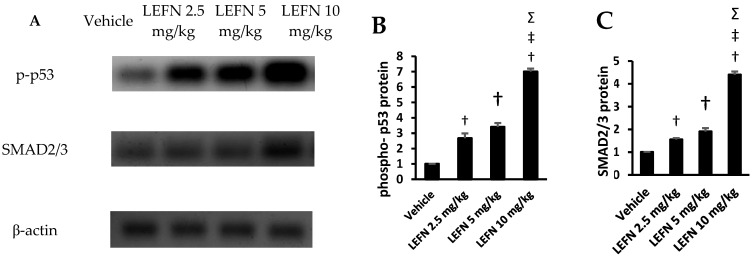
Western blot analysis for the target proteins. (**A**) The Western blot gels for p-p53, SMAD2/3, and SMA compared to β-ACTIN. (**B**,**C**) Column charts for the p-p53 and SMAD2/3 in the experimental groups. Data are mean ± SD. †: versus the vehicle group, ‡: versus the LEFN 2.5 mg/kg group, and Σ: versus the LEFN 5 mg/kg group at *p* < 0.05.

The Western blot gels are shown in Figure 10A for p-p53 and SMAD2/3. The mean values of p-p53 protein density are shown in Figure 10B, whereas the mean values for SMAD2/3 protein are shown in Figure 10C. Both proteins were significantly upregulated in the LEFN 10 mg/kg group compared to the other experimental groups.

## 4. Discussion

LEFN exhibits immunosuppressive and anti-inflammatory activity [55]. It is used as DMARD, since it inhibits the biosynthesis of pyrimidine and, in turn, inhibits the progression of disease [56]. Even though LEFN utility has increased during the past years, it is associated with many serious adverse effects affecting the immune system, hematological system, and hepatic system [57]. Moreover, this drug’s long half-life of 2 weeks delays the treatment of adverse reactions.

Drug-induced nephrotoxicity is a significant clinical challenge, particularly in immunocompromised patients. Although LEFN was approved to treat rheumatoid arthritis in 1998, renal impairment and severe interstitial nephritis were often reported as fatal side effects during the recommended therapeutic regimen [13,14,15,16]. Surprisingly, renal function is not monitored when receiving the recommended dose [2], and much uncertainty still exists about the exact mechanisms of renal injury induced by LEFN.

The present study confirms that LEFN induces dose-dependent kidney affection, which is identified in the form of elevations in renal function parameters (blood urea and BUN) and established histopathological abnormalities.

The results revealed that LEFN at a dose of 10 mg/kg markedly induced renal dysfunction, as indicated by higher levels of serum creatinine and BUN, and marked histopathological changes in the renal tissues. The second LEFN dose (5 mg/kg) induced moderate histopathological changes, with significant elevation in the BUN level only. Finally, the 2.5 mg/kg dose of LEFN was found to produce minimal histopathological abnormalities, with non-significant changes in serum creatinine or BUN.

The results are in agreement with the findings of [13,15,16,17], which showed the potential nephrotoxicity of LEFN. Hurtado et al. (2016) demonstrated that rapid deterioration of kidney function with severe tubulointerstitial nephritis confirmed by renal biopsy was proved in patients who received LEFN [16]. Chronic LEFN dosage was linked to IgA glomerulonephritis [58]. Furthermore, the combined therapy of methotrexate with LEFN was documented to cause acute kidney failure [59]. The elimination half-life of LEFN is about 2 weeks, and may be prolonged with renal impairment, which exerts more stress on kidneys [60]. To explain the relatively good response to 2.5 mg, the starting point of the injury may be the mitochondrial model of idiosyncrasy. The doses that saturate the capacity of the mitochondria end in drastic effects, whereas lower toxic doses produce off/on-like effects. A similar model has been seen in liver toxicity, as the toxicity is clearer in females and older age mice. Future studies can track such a hypothesis in Sod2 (+/−) mice to see if direct and mitochondrial causes can contribute to the pathology [61].

LEFN-induced hepatotoxicity has been reported in various studies [62,63,64]. The underlying pathogenesis involved in hepatotoxicity induced by LEFN is not fully elucidated. A liver signaling molecule, TGFβ, is involved in apoptosis, differentiation, and maturation [65]. Continuous inflammation of the liver and unrelieved injury leads to hepatic fibrosis. Hepatic stellate cell activation causes extracellular matrix deposition [66]. Even though a study conducted before has reported LEFN’s inhibitory effect on fibrosis of the liver, its cumulative dose has been reported to cause hepatic fibrosis in patients with rheumatoid arthritis [67]. TGFβ, by its paracrine and autocrine mechanisms, is involved in causing hepatic and lung fibrosis [68]. A review was conducted on renal toxicity associated with DMARDs used for the treatment of rheumatoid arthritis [69], and it was reported that LEFN exhibits kidney toxicity. Similarly, the current results showed an increment in renal TGFβ in mice who received LEFN, indicating that LEFN induces nephrotoxicity.

TGFβ is a group of growth factors promoting inflammation, growth, and differentiation. In mammals, there are three isoforms, ranging from TGFβ 1, 2, and 3. It is secreted as a precursor that is activated with multiple signaling pathways [70]. Progressive renal failure is characterized by renal fibrosis replacing active kidney cells. Whatever the cause of renal failure, the condition is associated with high TGFβ. The hypothesis behind that unfortunate organ failure is the activation of the TGFβ/Smad pathway initiating pro-fibrogenic cascade [71]. Experimental inhibition of TGF beta1 resulted in a reduction of the fibrosis process in the kidney. The TGFβ can activate the myofibroblasts and induce the extracellular matrix to proliferate via the Smad pathway and less common ones. The Smad track modifies the balance between fibrotic and anti-fibrotic situations. The TGFβ is controlled through epigenetic mechanisms [35]. In obstructive kidney fibrosis, it has been shown that TGFβ promotes the transition of pericytes to myofibroblasts [72]. The main source of this cytokine is macrophages actively working in the fibrotic area [73].

TGFβ1 has other functions than the induction of fibrosis; it has regulatory functions of apoptosis, cell proliferation, differentiation, and inhibition of the immune reaction. The latter function causes concerns about the use of TGF beta in treating progressive renal failure [74]. TGFβ via Smad3 induces a battery of genes involved in the fibrosis process, and indirectly inhibits the anti-fibrotic genes. There are clinical hopes that the anti-Smad strategy can be an alternative mechanism to overcome chronic progressive renal fibrosis [35].

Beside nephrotoxicity, two recent studies highlighted dose-dependent toxicity for the liver and the lungs of adult mice. The first one explored the hepatotoxicity, whereas the second one explored the pulmonary toxicity in albino mice.

The histopathological findings of LEFN on the liver showed that the pathologic score in the LEFN-treated groups was greater than the vehicle group. Further, results highlighted that LEFN dose-dependently raised the hepatic TLR4 immunostaining. The gene expression studies in liver tissues indicated significant over-expression of PI3K and TGFβ in LEFN-treated mice. An ELISA test confirmed that there was a significant elevation of TGFβ in mice treated with the different doses of LEFN [3].

A previous experimental study investigated the pulmonary toxicity of leflunomide in mice. The hematoxylin and eosin staining of lung specimens demonstrated fibrotic changes in the lungs of albino mice, which were dose-dependent; these results were confirmed by Masson’s trichrome staining as well. LEFN use for eight weeks resulted in lung injury, and there was a marked increase in lung injury when the dose of LEFN increased. Western blot analysis revealed an increase in α-SMA, vimentin, and collagen 1 in LEFN-treated animals; there was also a dose-dependent increase of the same [12]. In the later study, elevated levels of inflammatory markers (NLRP3 and interlukin-1) were found in dose-dependent pulmonary toxicities, together with architectural structural distortion [12].

## 5. Conclusions

The current results demonstrated, for the first time, detailed and comprehensive results about the nephrotoxicity of leflunomide in mice. Leflunomide produced dose-dependent pathologic changes in the kidneys, including leukocyte infiltration, glomerular degeneration, and tubular regeneration. We demonstrated that this toxicity included upregulation of TGFβ-mediated p53/Smad2/3 signaling, and induction of fibrosis. An improved understanding of LEFN-induced nephrotoxicity would have great implications in the prediction, prevention, and management of leflunomide-treated rheumatic patients, and may warrant further clinical studies for following up these toxidromes.

## Data Availability

Data are available from the authors upon request.
